# Solution Structure of Tubuliform Spidroin N-Terminal Domain and Implications for pH Dependent Dimerization

**DOI:** 10.3389/fmolb.2022.936887

**Published:** 2022-06-14

**Authors:** Megija Šede, Jēkabs Fridmanis, Martins Otikovs, Jan Johansson, Anna Rising, Nina Kronqvist, Kristaps Jaudzems

**Affiliations:** ^1^ Department of Physical Organic Chemistry, Latvian Institute of Organic Synthesis, Riga, Latvia; ^2^ Department of Biosciences and Nutrition, Neo, Karolinska Institutet, Huddinge, Sweden; ^3^ Department of Anatomy, Physiology and Biochemistry, Swedish University of Agricultural Sciences, Uppsala, Sweden; ^4^ Faculty of Chemistry, University of Latvia, Riga, Latvia

**Keywords:** spider silk, tubuliform spidroin, N-terminal domain, NMR structure, dimerization mechanism

## Abstract

The spidroin N-terminal domain (NT) is responsible for high solubility and pH-dependent assembly of spider silk proteins during storage and fiber formation, respectively. It forms a monomeric five-helix bundle at neutral pH and dimerizes at lowered pH, thereby firmly interconnecting the spidroins. Mechanistic studies with the NTs from major ampullate, minor ampullate, and flagelliform spidroins (MaSp, MiSp, and FlSp) have shown that the pH dependency is conserved between different silk types, although the residues that mediate this process can differ. Here we study the tubuliform spidroin (TuSp) NT from *Argiope argentata*, which lacks several well conserved residues involved in the dimerization of other NTs. We solve its structure at low pH revealing an antiparallel dimer of two five-α-helix bundles, which contrasts with a previously determined *Nephila antipodiana* TuSp NT monomer structure. Further, we study a set of mutants and find that the residues participating in the protonation events during dimerization are different from MaSp and MiSp NT. Charge reversal of one of these residues (R117 in TuSp) results in significantly altered electrostatic interactions between monomer subunits. Altogether, the structure and mutant studies suggest that TuSp NT monomers assemble by elimination of intramolecular repulsive charge interactions, which could lead to slight tilting of α-helices.

## Introduction

Orb weaving spiders stand out among silk producers found in nature as they can produce up to seven different types of silk, each tailored for a specific function. The main components of the spider silk threads are large proteins called spidroins. Major ampullate spidroin (MaSp) is the main component of dragline silk, the strongest and toughest of all spider silk types (Ayoub et al., 2007). Spider silk formed from minor ampullate spidroin (MiSp) is used for auxiliary spiral stabilization (Gosline et al., 1986; Colgin and Lewis, 1998). Aciniform spidroin (AcSp) is the main component of wrapping silk used to immobilize prey (Tremblay et al., 2015). Pyriform spidroin (PySp) makes up the attachment discs, which lash the joints of the web and attaches dragline silk to surfaces (Perry et al., 2010). Flagelliform spidroin (FlSp) forms capture spiral silk, which can extend up to 500% of its length (Eisoldt et al., 2011). Aggregate spidroin (AgSp), coats the FlSp threads and is used as a glue to capture prey on the web (Opell and Hendricks, 2010; Collin et al., 2016). Tubuliform spidroin (TuSp) forms the outer layer of spider egg cases and protects the eggs from the external environment (Tian and Lewis, 2005; Eisoldt et al., 2011).

Each spidroin is produced in a specific gland. The major ampullate gland, which has been studied most extensively, has a long, winding, and narrow tail, a wider ampulla or sac, and an S-shaped narrowing duct connected to the sac via a funnel (Andersson et al., 2013). After secretion in the tail segment, spidroins are held in the ampulla at up to 50% (w/w) concentrations (Hijirida et al., 1996) and upon silk spinning, pulled through the tapered duct. Here, changes in the surrounding environment conditions (i.e., reduced pH, altered ion composition, increased partial CO_2_ pressure) and increasing shear forces cause them to assemble and form solid threads (Knight and Vollrath, 2001; Rousseau et al., 2004; Andersson et al., 2014; Sparkes and Holland, 2017, 2019). The tubuliform gland, on the other hand, is long, noodle-shaped, and smooth without a distinguishable ampulla-shaped storage sac (Chaw and Hayashi, 2018). Also, the conditions in the tubuliform gland are not known.

The conditionally high solubility and regulation of spider silk formation is mediated by two conserved spidroin terminal domains–the N-terminal domain (NT) and the C-terminal domain (CT) (Askarieh et al., 2010; Hagn et al., 2010), whereas the highly variable central repetitive domain (Rep), is responsible for the silk properties (Gosline et al., 1999). MaSp NTs from different spider species were early on found to be pH sensitive (Gaines et al., 2010; Landreh et al., 2010). In the ampulla at pH > 7 the NT forms a monomeric five-helix bundle showing a distinct dipolar distribution of charged amino acids (Askarieh et al., 2010; Jaudzems et al., 2012). Under these conditions, it likely promotes solubility of the spidroin Rep domain by forming the shell of micelle-like particles with the aggregation-prone regions sequestered in their core (Kronqvist et al., 2017). As spidroins are pulled through the spider silk duct, the pH is reduced to <5.7 (Andersson et al., 2014), which causes sequential protonation of a cluster of glutamic acids on the NT’s surface. This disrupts several charge interactions leading to movement of α-helices, relocation of a wedged W10 side chain from a buried to a surface exposed conformation, and subsequent dimerization of the domain. The NT dimerization results in firmly interconnected spidroins (CT is dimeric already during storage), which ensure propagation of pulling forces during the silk fiber formation (Kronqvist et al., 2014; Ries et al., 2014).

The pH dependent dimerization mechanism seems conserved among NTs from different silk types and species (Heiby et al., 2017); however, the exact structural details are divergent (Otikovs et al., 2015). Through studies of site-directed mutants bearing glutamate to glutamine substitutions, the residues E79, E84 and E119 were identified to be protonated in the *Euprosthenops australis* MaSp NT dimer (Kronqvist et al., 2014). In *Araneus ventricosus* MiSp NT, E84 is substituted by a serine and the nearby E73 was instead found to be protonated (Otikovs et al., 2015). Similar mutants were investigated for *Nephila clavipes* FlSp NT and *Latrodectus hesperus* MaSp NT, however, the exact carboxylates to be protonated could not be identified (Bauer et al., 2016; Sarr et al., 2022). AcSp from *Nephila antipodiana* displays a very different charge distribution on the protein surface, nevertheless it still forms dimers at low pH and in presence of physiological salt concentrations (Chakraborty et al., 2020).

Three other MaSp NT mutants have been investigated to characterize its monomeric conformation. Residues D40 and K65 located at opposite tips of the NT molecular dipole form an intermolecular salt bridge in the dimer and were proposed to mediate initial monomer association (Schwarze et al., 2013; Kronqvist et al., 2014). The residue charge reversal in the mutant NT_D40KK65D_ resulted in disturbed dipolar interactions between NT monomers and abolished its pH sensitivity (Kronqvist et al., 2017). This variant shows excellent solubility-enhancing properties and has been applied as solubility tag for recombinant production of aggregation-prone proteins and peptides (Kronqvist et al., 2017, 2022; Sarr et al., 2018; Abelein et al., 2020). Another pH independent NT monomer was prepared by replacing an alanine residue in the middle of the dimer subunit interface with an arginine, thereby preventing self-association through charge repulsion (Jaudzems et al., 2012). In the third mutant, all six methionine residues in the protein core were replaced by leucines, which significantly reduced protein plasticity and abolished the movement of α-helices necessary for NT dimerization (Heiby et al., 2019).

**FIGURE 1:**
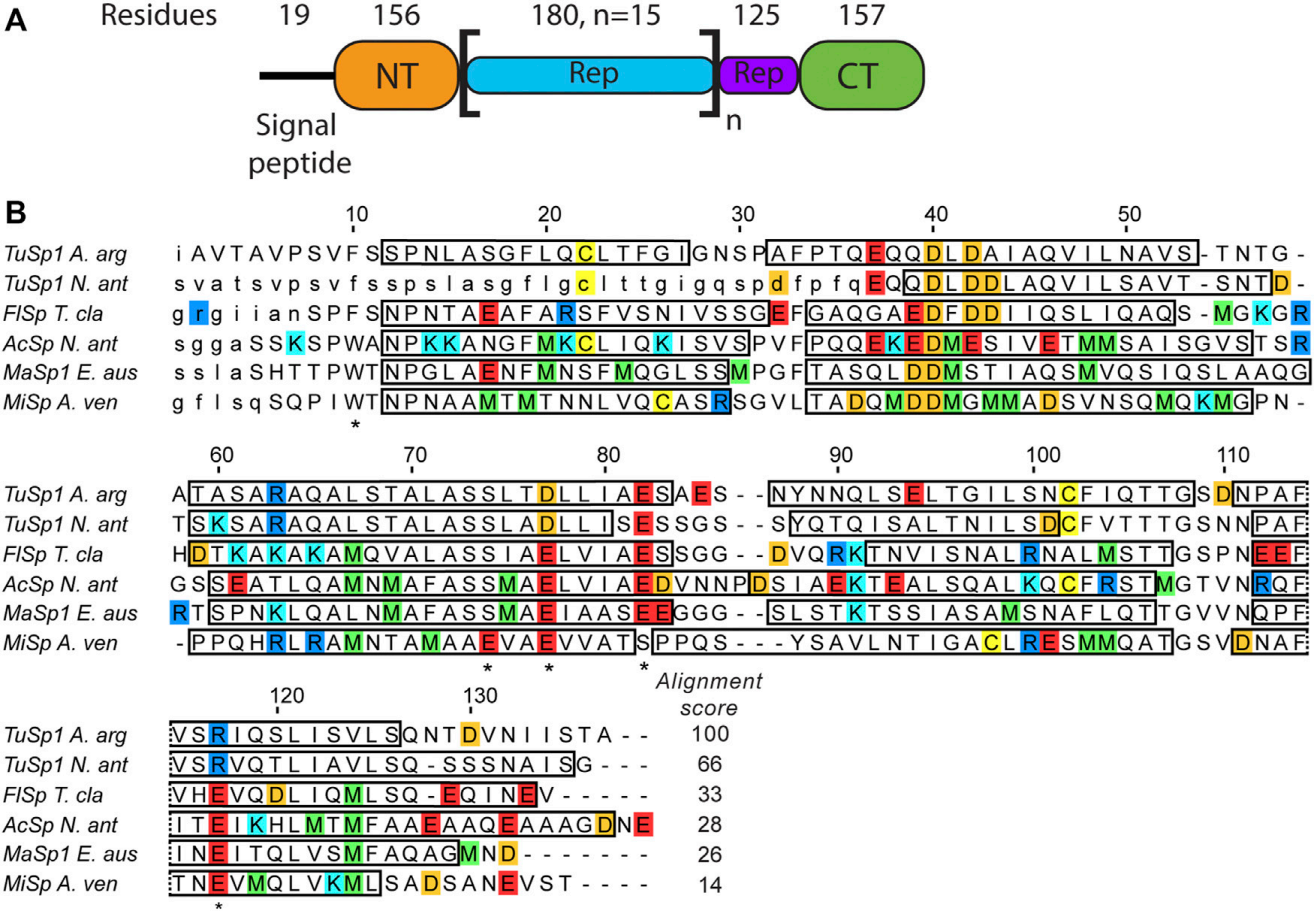
**(A)** *Argiope argentata* TuSp domain architecture (Chaw et al., 2018). The number of residues for NT is different from the construct used in this study because it includes ∼20 non-conserved residues linking it to the Rep region. **(B)** Amino acid sequence alignment of spidroin NTs with known tertiary structures. Included are NTs from *Argiope argentata* TuSp (PDB ID 6TV5), *Nephila antipodiana* TuSp (PDB ID 2K3Q), *Nephila clavipes* FlSp (PDB ID 7A0O), *Nephila antipodiana* AcSp (PDB ID 7BUT), *Euprosthenops australis* MaSp (PDB ID 2LTH) and *Araneus ventricosus* MiSp (PDB ID 2MX9). Locations of α-helices are indicated by rectangles and amino acids excluded from the expression vector are in small caps. Residues protonated upon dimer formation (E79, E84, E119 in MaSp and in MiSp E73, E79 and E119) and W10/F10 are marked with an asterisk below the sequence. Arginines are blue, lysines are cyan, glutamic acids are red, aspartic acids are orange, methionines are green and cysteines are yellow. ClustalW alignment score to *E. australis* MaSp NT is shown at the end of each sequence.

The structures of all TuSp domains from *Nephila antipodiana* were the first published silk protein structures (Lin et al., 2009). The TuSp NT structure was determined by solution NMR in presence of 100 mM dodecylphosphocholine (DPC) to avoid protein aggregation and using a protein construct, which lacked the first 36 amino acids. The structure shows a four-helix bundle, which differs from the more recent structures of other NTs comprising five helices (Figure 1). The amino acid sequence of TuSp NT also displays some notable differences in comparison to the consensus sequence of other NTs. The E119 of MaSp NT involved in the protonation during dimerization is replaced by an arginine. Furthermore, the sequence contains no methionines, which were found to enable the helical reorganization during MaSp NT dimerization. Additionally, in TuSp NT (similarly to FlSp NT), W10 is replaced by a phenylalanine.

Considering the additional mechanistic insights gained from the recent studies of MaSp, MiSp and FlSp NT and the contrasting structural data of TuSp NT, we decided to re-investigate its structure and possible dimerization mechanism using a protein construct that comprises the complete conserved domain sequence from *Argiope argentata*. We use nuclear magnetic resonance (NMR) to characterize the TuSp NT conformation at pH > 7 and to determine its structure at pH 5.5. Next, several methods including NMR, size exclusion chromatography (SEC), and circular dichroism (CD) are used on the wild type (wt) protein as well as site-directed mutants to investigate the dimerization mechanism of TuSp NT.

## Methods

### Site-Directed Mutagenesis of *A. argentata* TuSp NT

pET-32a-HisTrxHisNT plasmid containing wt *A. argentata* TuSp 1 NT sequence and pET-28a-HisNT containing TuSp NT_E37QE82Q_ and TuSp NT_E37QE82QE85Q_ were ordered from BioCat GmbH (Heidelberg, Germany). For expression of TuSp NT, TuSp NT_A70E_, and TuSp NT_A70R_ the pET-32a vector was used. TuSp NT_D40RR63D_, TuSp NT_E37QE82Q_, TuSp NT_E37QE82QE85Q_, and TuSp NT_E37QE82QE85QD130N_ were expressed by using the pET-28a vector (Supplementary Figure S1 for the amino acid sequences). To obtain TuSp NT_A70E_, TuSp NT_A70R_ and TuSp NT_E37QE82QE85QD130N_, pET-32a-HisTrxHisNT plasmid containing TuSp NT sequence or pET-28a-HisNT containing TuSp NT_E37QE82QE85Q_ were subjected to point mutagenesis using Phusion site-directed mutagenesis kit (ThermoFisher Scientific, United States). At first, two PCR reactions were carried out in parallel with forward and reverse primers in separate tubes. After PCR, the two products were combined in a 1:1 ratio and another thermocycle was run. PCR products were digested with DpnI, and purity of plasmids was checked on 1% agarose gel. The plasmids were used to transform chemically competent *E. coli* XL1*-*Blue cells by heat shock, which was followed by plasmid extraction and sequence verification.

### Protein Expression and Purification

The plasmids were used for heat shock transformation of chemically competent *E. coli* BL21 (DE3) cells. Overnight cultures were inoculated at a ratio of 1:100 into LB medium containing 50 μg/ml kanamycin (pET-28a plasmids) or 100 μg/ml ampicillin (pET-32a plasmids) and cells were further grown at 37°C and 180 rpm to an OD_600_ of 0.6. Expression was induced by addition of isopropyl β-D-1-thiogalactopyranoside (IPTG) to a final concentration of 0.5 mM and cultures were further incubated overnight at 25°C. The cells were harvested by centrifugation at 7000 g for 10 min and stored at −20°C.

Cells were thawed and resuspended in the immobilized metal-affinity chromatography (IMAC) loading buffer (20 mM Tris-HCl, 8 M urea, 300 mM NaCl, 15 mM imidazole, 1 mM DTT, pH 8.0). The cells were sonicated, and the lysate was cleared by centrifugation at 20000 g for 40 min. The supernatant was then loaded on a 2 × 5 ml HisTrap HP column (Cytiva). Unbound proteins were washed off with 5 column volumes of loading buffer. Bound proteins were eluted in 2 ml fractions using 500 mM imidazole, 20 mM Tris-HCl, 8 M urea, 300 mM NaCl, 1 mM DTT, pH 8.0. The absorbance at 280 nm was measured for each fraction and protein-containing fractions were pooled and dialyzed against 20 mM Tris-HCl, 300 mM NaCl, 1 mM EDTA, 1 mM DTT, pH 8.0 at 4°C. Complete refolding of the protein was verified by NMR. Fusion proteins were cleaved with tobacco etch virus (TEV) protease (ratio of 1:10–1:20, enzyme to substrate, w/w) during dialysis against TEV reaction buffer (50 mM Tris-HCl, 1 mM DTT, 0.5 mM EDTA, pH 8.0) at 4°C. After the cleavage, sample was loaded on the HisTrap HP column to remove the cleaved fusion tag and unbound target protein was collected. Protein was further purified by gel filtration with a HiLoad 16/600 Superdex 75 pg size exclusion column (Cytiva). The concentration of protein was determined by using the Pierce Bicinchoninic Acid (BCA) protein assay kit (Thermo Fisher Scientific, United States) according to the manufacturer’s recommendations. The correct size of protein was confirmed by SDS-PAGE using 4–12% Bis-Tris polyacrylamide gel (ThermoFisher Scientific, United States), stained with Coomassie brilliant blue dye.

For production of ^15^N- and ^13^C, ^15^N-labeled samples the same procedure was used except that M9 minimal medium containing ^15^NH_4_Cl and ^13^C-glucose as the sole sources of nitrogen and glucose, respectively, was used.

### NMR Spectroscopy

The protein sample used for NMR structure determination was concentrated to approximately 1 mM in 20 mM sodium acetate-d_3_ (NaOAc-d_3_), pH 5.5, 20 mM NaCl, 0.03% (w/v) NaN_3_, 5% D_2_O (v/v) buffer. NMR spectra were acquired at 298 K on a Bruker Avance Neo 600 MHz spectrometer equipped with a TCI z-gradient cryoprobe. For backbone assignment, 3D HNCA, 3D HNCO, 3D CBCA(CO)HN, and 3D HN(CA)CO spectra were acquired. For side chain assignment and structure determination, 3D [^1^H,^1^H]-nuclear Overhauser spectroscopy (NOESY)-^15^N-heteronuclear single-quantum correlation (HSQC), 3D [^1^H,^1^H]-NOESY-^13^C (aliphatic)-HSQC, 3D [^1^H,^1^H]-NOESY-^13^C (aromatic)-HSQC spectra were acquired with a mixing time of 80 ms. Chemical shifts were referenced internally to the residual water signal at 4.77 ppm relative to 4,4-dimethyl-4-silapentane-1-sulfonic acid (DSS).

For recording of 2D^15^N-^1^H HSQC NMR spectra, ^15^N-labeled samples were prepared in either 20 mM NaOAc-d_3_, 20 mM NaCl, pH 5.5 or 20 mM sodium phosphate (NaPi), 300 mM NaCl, pH 7.2 buffers. The spectra were acquired using either Bruker Avance III HD 800 MHz spectrometer equipped with an TXI z-gradient room temperature probe or Bruker Avance Neo 600 MHz spectrometer equipped with a TCI z-gradient cryoprobe.

The molecular weight of TuSp NT at pH 5.5 was estimated using 2D ^1^H-detected ^15^N relaxation experiments. The relaxation data were recorded on the Bruker Avance III HD 800 MHz spectrometer equipped with an TXI z-gradient room temperature probe. Spectra were processed in TopSpin 4.1.1 and signal intensities were measured with CARA 1.8.4.2 (Keller, 2004). Residue-specific protein backbone amide ^15^N NMR R_1_ and R_2_ relaxation times were calculated from the peak intensities using two-parameter exponential fit model in Relax 4.0.3 (d’Auvergne and Gooley, 2008). Seven T_1_ (20–2000 ms) and seven T_2_ (10–250 ms) delays were used for the recording of the decay curves. Protein rotational correlation time (τ_c_) was calculated using the equation

$$\tau_{c}\approx \frac{1}{4{\text{πv}}_{N}}\sqrt{6\frac{T_{1}}{T_{2}}-7}$$

(Kay et al., 1989), where ν_N_ is the ^15^N resonance frequency (in Hz), T_1_ is average longitudinal and T_2_ average transverse relaxation time value of rigid protein regions.

### NMR Structure Calculation

All acquired spectra were processed using TopSpin 4.1.1 software. The assignment of protein backbone and side chain chemical shifts was performed with CARA 1.8.4.2 (Keller, 2004). The peak lists for dimeric structure calculation were acquired from an initial monomeric structure calculation using UNIO-ATNOS/CANDID 2.0.2 (Herrmann et al., 2002a, 2002b; Serrano et al., 2012) in conjunction with CYANA 2.1 (Güntert et al., 1997). Subsequent manual inspection of the NOESY spectra allowed the identification of 11 unambiguous unique intermolecular contacts that were used as distance constraints with an upper limit of 5 Å in the following structure calculations. Notably, these contacts are only a small fraction of all intermolecular contacts found during automated NOE assignment and structure calculation. The dimeric structure calculation was done using seven iterations of CYANA protocol, starting with 100 random conformers that were subjected to simulated-annealing with 10,000 steps of torsion-angle molecular dynamics. The structural statistics were further improved by applying TALOS+ angle restraints. For the final structure calculation, a homology model obtained from SwissProt was used as a starting conformer in the first cycle of the calculation to improve convergence. The 20 conformers with the lowest residual CYANA target-function values after CYANA cycle 7 were energy minimized in explicit water using CNS (Brunger, 2007).

### Size Exclusion Chromatography

Size exclusion chromatography on the purified TuSp NT and its variants was performed using an Äkta Purifier 10 system. 50 µl of purified protein at a concentration of 3 mg/ml were loaded onto a Superdex 75 Increase 10/300 GL size exclusion column (Cytiva) previously equilibrated in the relevant buffer (20 mM Tris-HCl, 300 mM NaCl, pH 8.0 or 20 mM NaOAc, 20 mM NaCl, pH 5.5). The molecular weight calculation was performed according to the calibration found in the column manual, where conalbumin (75 kDa) was measured to elute at 8.7 ml, ovalbumin (44 kDa) at 9.65 ml, carbonic anhydrase (29 kDa) at 11.2 ml, ribonuclease (13.7 kDa) at 13.3 ml and aprotinin (6.5 kDa) at 16.1 ml.

### Circular Dichroism Spectroscopy

CD experiments were performed on a Jasco J-1500 CD spectrometer using 300 µl cuvettes with 1 mm path length. All measurements were acquired in 20 mM sodium phosphate buffer at pH 8.0 or pH 5.5 at 10 µM protein concentration. Spectra were recorded from 260 to 185 nm, first at 25°C, then after heating for 15 min at 95°C and finally after cooling down to 25°C. 5 scans were recorded for each temperature to calculate an average spectrum. For temperature melting curves, the CD intensity at 222 nm was monitored between 25 and 95°C with 1°C/min increase.

## Results

### NMR Sample Preparation

*A. argentata* TuSp NT with N-terminal His-Trx fusion tag was expressed as insoluble inclusion bodies in *E. coli* BL21 (DE3). Following solubilization in 8 M urea, the protein was purified using IMAC and refolded by dialysis against Tris buffer at pH 8 and 4°C. The His-Trx solubility tag was subsequently cleaved with TEV protease. The cleaved protein was once again passed through an IMAC column to remove the tag and further purified by gel filtration in presence of 300 mM NaCl to isolate monomeric TuSp NT.

**FIGURE 2:**
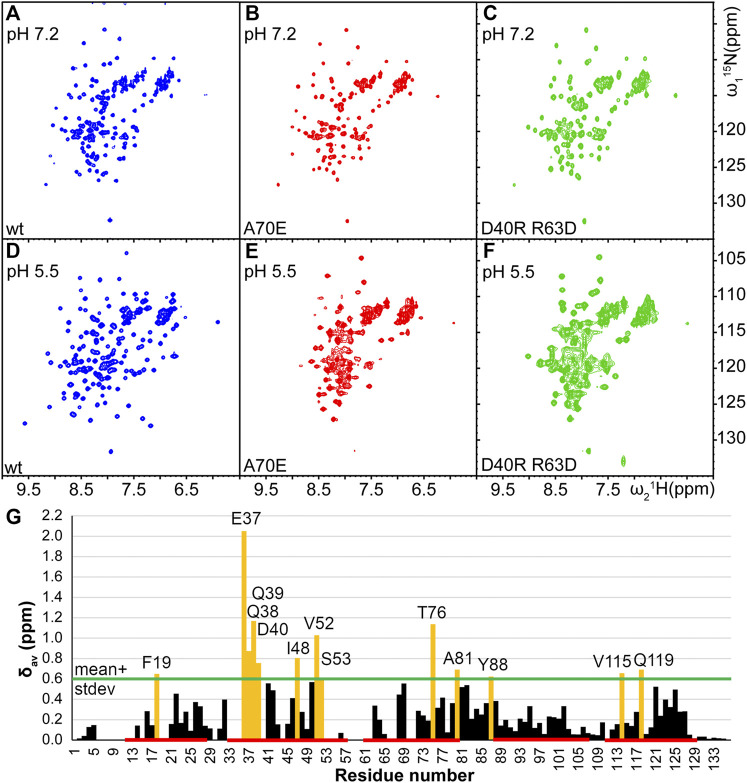
Comparison of ^15^N-^1^H HSQC spectra of TuSp NT (blue,**A** and **D**) and its potential monomeric mutants TuSp NT_A70E_ (red,**B** and **E**), and TuSp NT_D40RR63D_ (green,**C** and **F**) in 300 mM NaCl, 20 mM NaPi, pH 7.2 and 20 mM NaCl, 20 mM NaOAc, pH 5.5 respectively. **(G)** Averaged backbone amide ^15^N and ^1^H chemical shift differences $\Delta \delta_{av}=\sqrt{{\left(0.1\Delta \delta_{N}\right)}^{2}+{\left(\Delta \delta_{H}\right)}^{2}}$ between TuSp NT_A70E_ at pH 7.2 and wt TuSp NT dimer at pH 5.5. Residues showing chemical shift differences larger than mean plus one standard deviation are highlighted in yellow and identified. Locations of helices are indicated with a red line.

In order to determine optimal solution conditions for the NMR structure determination, the protein was dialyzed against several different buffers with pH between 5.5 and 8, and variable amount of salt. The spectral quality was evaluated by ^15^N-^1^H HSQC NMR spectroscopy. A high-quality spectrum was obtained only at low pH conditions corresponding to the spider’s spinning duct (20 mM NaOAc at pH 5.5, 20 mM NaCl), as indicated by ∼130 well separated peaks with similar intensities (Figure 2). At high pH conditions as in the ampulla (20 mM phosphate at pH 7.2 or 20 mM Tris-HCl at pH 8) and in presence of 0.3–1 M NaCl the number of well separated observed peaks was reduced to ∼110 suggesting that the protein is in conformational exchange.

### NMR Analysis of TuSp NT Monomer at High pH

As described above, at pH > 7 and in presence of high salt concentrations (i.e. conditions, where MaSp NT is monomeric) TuSp NT showed a reduced number of HSQC NMR signals. To stabilize its monomeric conformation, three mutants were prepared by introducing single or double-site mutations, which prevent dimerization of MaSp NT (Jaudzems et al., 2012; Kronqvist et al., 2017). Firstly, A70 located in the middle of the dimerization interface was replaced with glutamic acid or arginine residues that could prevent dimerization through electrostatic and steric repulsion (mutants TuSp NT_A70E_ and TuSp NT_A70R_). Secondly, the molecular dipole of each subunit important for the initial association of monomers was disrupted by charge reversal of the residues D40 and R63 (mutant TuSp NT_D40RR63D_). TuSp NT_A70E_ and TuSp NT_D40RR63D_ variants could be produced in sufficient quantities for NMR experiments using the same protocol as for the wt protein, whereas TuSp_A70R_ could not be properly refolded. The inability to refold TuSp NT_A70R_ is likely associated with formation of a new electrostatic interaction that is incompatible with the correct fold of the protein.

The HSQC spectra of TuSp NT_A70E_ and TuSp NT_D40RR63D_ at both, pH 7.2 and pH 5.5, were similar to the spectrum of wt TuSp NT at pH 7.2 suggesting that dimerization was prevented. However, for TuSp NT_A70E_ at pH 5.5 and for TuSp NT_D40RR63D_ at both pH values the spectra also showed peak broadening implying that the monomeric conformation was not fully stabilized (Figure 2). The HSQC spectrum of TuSp NT_A70E_ showed slight improvement in spectral quality at pH 7.2 and 300 mM NaCl compared to wt protein as manifested by a more uniform peak intensity distribution. Therefore, we recorded and analyzed 3D triple-resonance spectra for chemical shift assignment of its backbone resonances. The assignment was obtained for 104 out of 136 residues and analysis of secondary chemical shifts indicated presence of five α-helices (Supplementary Figure S2). The residues 7–16, 20–21, 34–36, 54–63, 67–68, 71–72 corresponding largely to the N-terminal loop region, N-terminal part of first helix, loop between second and third helix as well as first half of third alpha helix could not be assigned. Mapping of these regions on the MaSp NT monomer structure showed that they cluster in the proximity of W10 (Supplementary Figure S2), suggesting that F10 in TuSp NT could be involved in a conformational exchange process, which results in disappearance of ∼20 backbone amide signals at pH 7.2.

### NMR Structure of TuSp NT Dimer at Low pH

**FIGURE 3:**
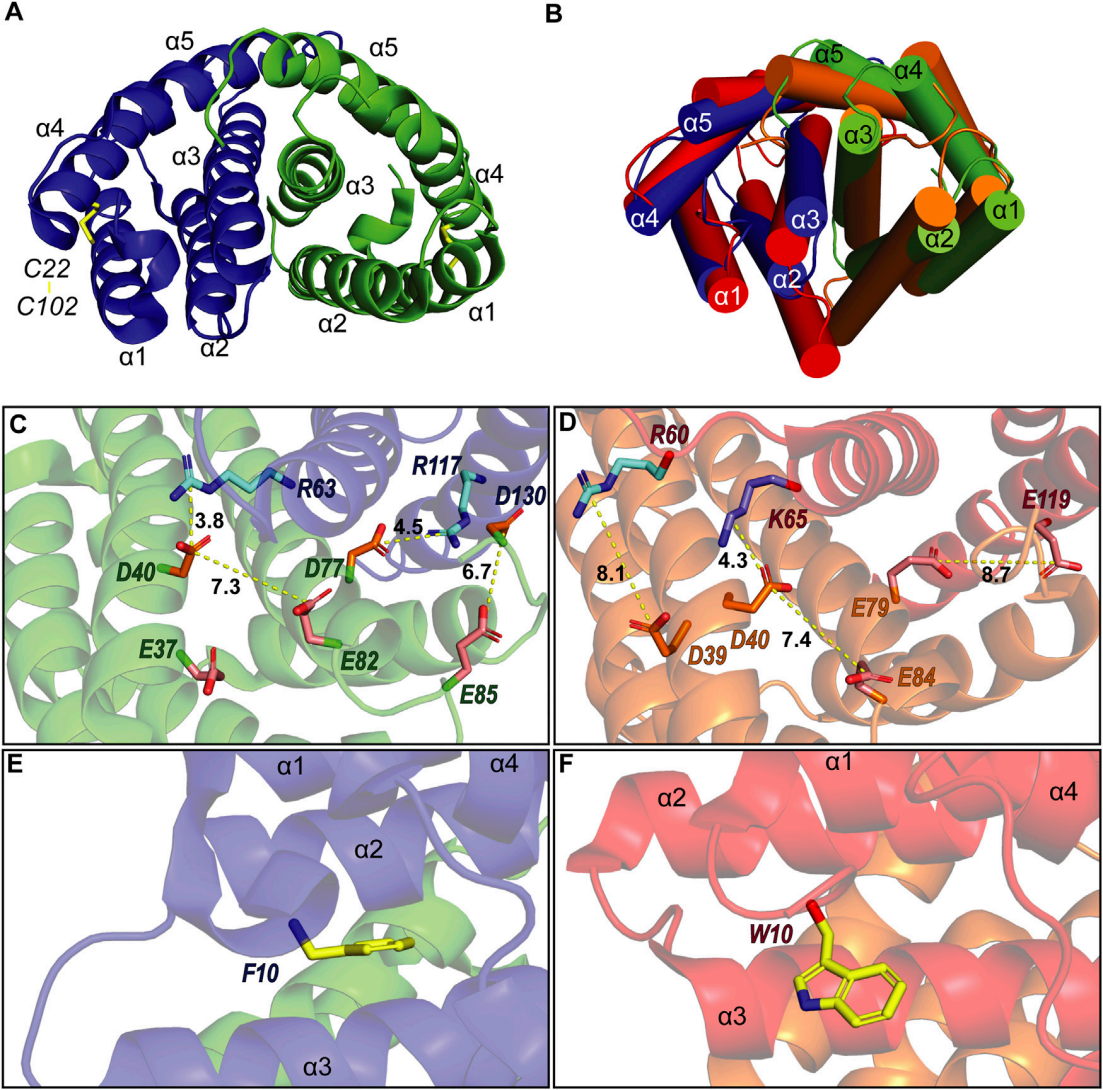
**(A)** Structure of *A. argentata* TuSp NT dimer (PDB ID 6TV5) with the two subunits shown in blue and green and their helices numbered. The intramolecular disulfide is highlighted with yellow stick representation. **(B)** Superposition of the structures of TuSp NT and *E. australis* MaSp NT (PDB ID 2LTH, red) dimers. α-helices are shown as cylinders. **(C)** and **(D)** shows charge interactions between residues on the subunit interface for *A. argentata* TuSp **(C)** and *E. australis* MaSp **(D)** NT, respectively. Aspartic acid side chains are colored orange, glutamic acids are pink, arginines are cyan and lysines are blue. **(E)** and **(F)** show the orientation of F10/W10 side chain within each structure (yellow).

To verify that TuSp NT is in a dimeric conformation at pH 5.5, ^15^N NMR relaxation measurements were performed. The bulk T_1_ and T_2_ relaxation times were determined to be 605 and 17 ms affording an estimated molecular tumbling correlation time of about 17 ns (assuming molecular isotropic motion), which matches well with the expected molecular mass of the TuSp NT dimer (28 kDa). The backbone assignment was performed using standard triple-resonance 3D experiments (Supplementary Figure S3) and distance restraints were obtained from three 3D^15^ N/^13^C-resolved NOESY-HSQC spectra. The dimeric structure of TuSp NT was calculated from 3663 NOEs including 84 intermolecular restraints (Figure 3, Table 1 for structural statistics). The folded part of each subunit is composed by residues 7–127 and consists of five α-helices (Figure 3A). The first and fourth α-helices are covalently linked through a disulfide bond between C22 and C102, which is confirmed by the cysteine ^13^C_β_ chemical shift values of ∼37 ppm corresponding to the oxidized state. The dimer interface is formed by the second, third and fifth α-helices. The overall structure is an antiparallel homodimer and strongly resembles the dimeric structures of other spidroin NTs (Figure 3B) displaying a backbone RMSD of 2.11 Å to MaSp NT (over 121 residues). However, the helical orientations are slightly different, in particular, the C-terminus of α2 is tilted towards α1 and α3.

**TABLE 1:** Input for the structure calculation and structural statistics for the energy-minimized NMR structure of TuSp NT at pH 5.5.

Distance constraints	
Total NOE	3663
Intra-residue (|i-j| = 0)	1010
Inter-residue	2653
Sequential (|i-j| = 1)	1077
Medium-range (|i-j| < 4)	760
Long-range (|i-j > 5)	732
Intermolecular	84
Violations (mean and s.d.)	
Distance constraints (Å)	0.0103 ± 0.0010
Max. distance constraint violation (Å)	0.37 ± 0.17
Number ≥ 0.1 Å	31 ± 5
PARALLHDG force field energies (kcal/mol)	
Total	−9987 ± 96
van der Waals	−1106 ± 33
Electrostatic	−10597 ± 102
Average pairwise r.m.s.d.* (Å)	
Heavy atoms (residues 7–55, 60–127)	0.92 ± 0.06
Backbone (residues 7–55, 60–127)	0.64 ± 0.07
Ramachandran plot statistics (%)	
Most favored regions	89.0
Additional allowed regions	10.0
Generously allowed regions	0.5
Disallowed regions	0.5^a^

a The residues found in the disallowed regions are A84 (in 6 conformers), E85 (10 conformers), D130 (2 conformers), N132 (1 conformer).

As described in the introduction, charged residues play an important role in the dimerization mechanism of the NTs. Of the three glutamates (E79, E84, and E119) that become protonated during MaSp NT dimerization, only E84 is conserved in TuSp NT (as E82). This residue shows a similar side chain orientation in the TuSp NT dimer, pointing towards D40 of the same subunit, with which it forms a characteristic handshake interaction in the MaSp NT dimer structure (Figures 3C,D). However, E79 and E119 in MaSp NT are replaced by D77 and R117 in TuSp NT. Since these residues also show similar side chain orientations, an intermolecular salt bridge is formed in the TuSp NT dimer. This finding implies a different mechanism for pH dependent dimerization of TuSp NT, not involving protonation of D77 (and R117). In the vicinity of TuSp NT E82 is another, non-conserved glutamate residue E37, which may be important for elevating the pK_a_ value of E82. Two other acidic residues, E85 and D130 from the same subunit, are close in space and pointing towards each other in some of the NMR conformers (Figure 3C). Thus, they could potentially be involved in the pH sensitivity via stabilization of a dimerization-capable conformation upon protonation. The TuSp NT dimer is further stabilized by the conserved D40-R63 salt bridge (D40-K65 in MaSp NT). The F10 residue is buried in a pocket between the helices and in contrast to MaSp NT W10 is not solvent exposed in the dimer but assumes a conformation more like that in MaSp NT monomer (Figures 3E,F).

Comparison of the assigned chemical shifts of the TuSp NT dimer and TuSp NT_A70E_ (Figure 2G) showed that the largest differences are clustered in helix α2, C-terminal part of helix α3, the loop between α3 and α4 as well as helix α5. As expected, all these regions are located close to the dimer subunit interface. The acidic amino acid residues that show largest chemical shift changes are E37, D40, D42, and E82.

### Dimerization Analysis

Unlike MaSp NT, the TuSp NT sequence does not contain the W10 residue, which prevents monitoring of the monomer-to-dimer transition by tryptophan fluorescence (Kronqvist et al., 2014; Otikovs et al., 2015). Hence, we used HSQC NMR spectroscopy, size exclusion chromatography (SEC) and circular dichroism (CD) to evaluate the dimerization behavior of TuSp NT and three mutants, in which the potentially titratable glutamate/aspartate residues were replaced by the respective amides. Based on the structure analysis of the TuSp NT dimer and chemical shift comparison between TuSp NT_A70E_ and TuSp NT dimer, a double, a triple and a quadruple mutant was designed, and the mutations were introduced by site-directed mutagenesis (mutants TuSp NT_E37QE82Q_, TuSp NT_E37QE82QE85Q_ and TuSp NT_E37QE82QE85QD130N_). The monomeric mutants TuSp NT_A70E_ and TuSp NT_D40RR63D_ were also included as controls.

Comparison of HSQC spectra of TuSp NT at pH 7.2 and 5.5 with the corresponding spectra of the three mutants indicated high structural similarity at pH 5.5 (corresponding to dimeric state) but less similar conformations at pH 7.2 (Supplementary Figure S4). However, all the mutants retained pH responsiveness as the spectra at pH 7.2 and 5.5 differed significantly. This result suggests that some other amino acid residue than the mutated ones or some of the investigated residues but in different combinations are protonated in this pH interval.

**FIGURE 4:**
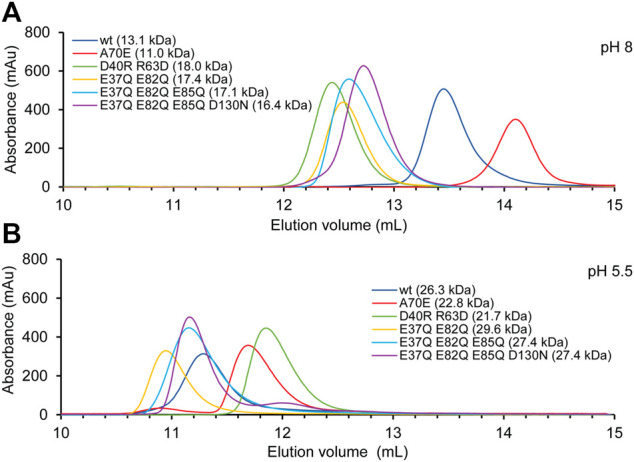
TuSp NT dimerization analysis by size exclusion chromatography performed in **(A)** 20 mM Tris-HCl, 300 mM NaCl, at pH 8 and **(B)** 20 mM NaOAc, 20 mM NaCl at pH 5.5.

SEC was performed in presence of 300 mM NaCl at pH 8 and 20 mM NaCl at pH 5.5. The experiments with TuSp NT showed that it migrates with an apparent molecular weight of 13.1 kDa at pH 8 and of 26.3 kDa at pH 5.5 (Figure 4). These values agree well with the calculated molecular weights of monomers (14.1 kDa) and dimers (28.1 kDa). The apparent molecular weight of TuSp NT_A70E_ at pH 8 was 11.0 kDa. As its HSQC spectrum showed no signs of partial unfolding, the reduced apparent molecular weight could suggest that this mutation indeed stabilizes the monomeric state. However, at low pH TuSp NT_A70E_ eluted with an apparent molecular weight of 22.8. kDa indicating that it can still dimerize. TuSp NT_D40RR63D_ eluted with apparent molecular weights of 18.0 (pH 8) and 21.7 kDa (pH 5.5) suggesting an intermediate state at both pHs, which is in line with presence of both species and with data from HSQC NMR experiments. The three acidic residue mutants TuSp NT_E37QE82Q_, TuSp NT_E37QE82QE85Q_, TuSp NT_E37QE82QE85QD130N_ displayed elution volumes consistent with an intermediate state at pH 8 and dimer state at pH 5.5. Thus, all three mutants kept responsiveness to pH suggesting that we failed to create a constitutive dimer.

**FIGURE 5:**
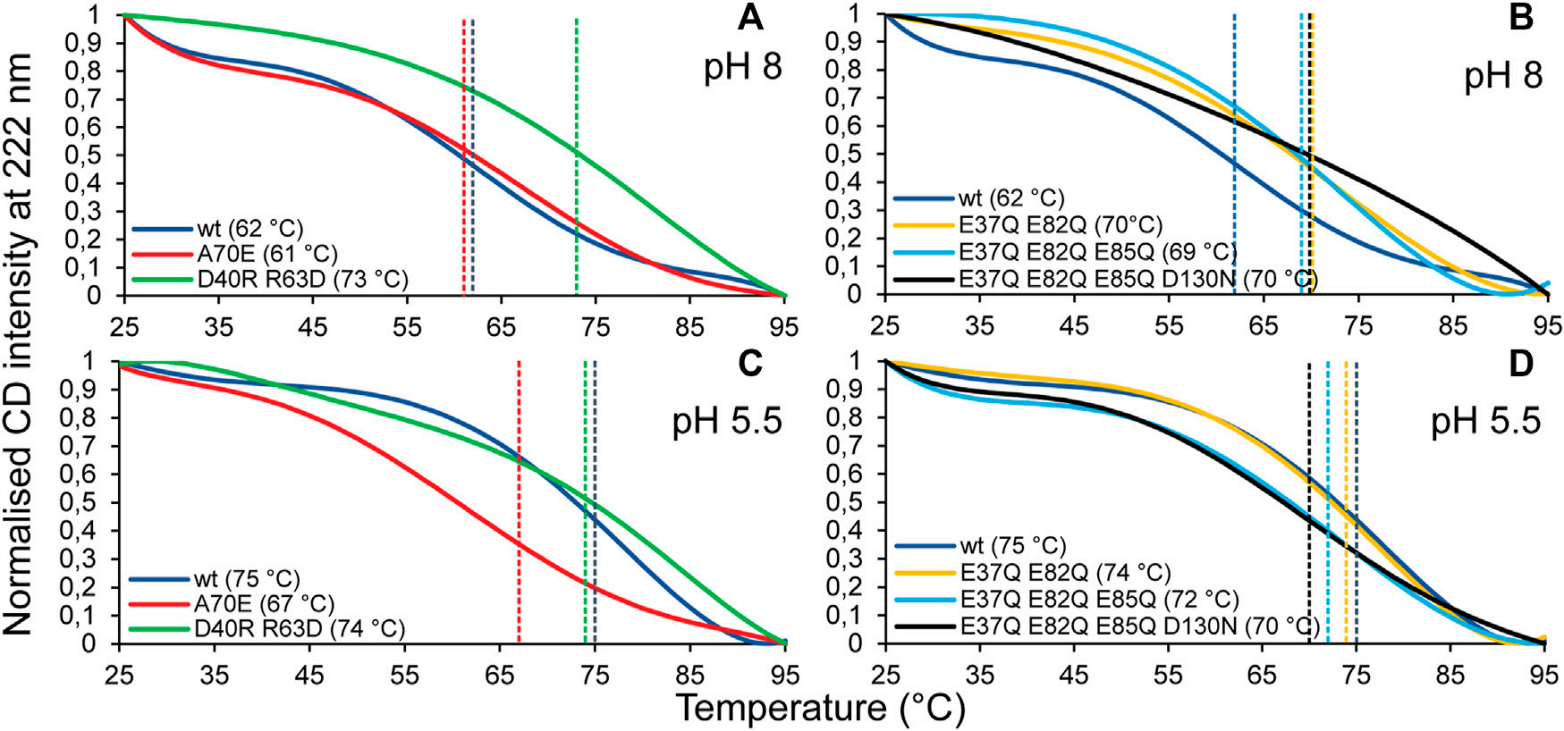
CD melting temperature (T_m_) scans of TuSp NT in 20 mM NaPi, pH 8 **(A,B)** and in 20 mM NaPi, pH 5.5 **(C,D)**. The CD signal intensity at 222 nm was monitored between 25 and 95°C with 1°C/min increase.

CD experiments were carried out to evaluate the refolding capacity and thermal stability of the different TuSp NT variants at pH 8 and pH 5.5. At 25°C the CD spectra of all analyzed proteins showed a maximum at 190 nm and double minima at 208 and 222 nm irrespective of the sample pH, consistent with α-helical secondary structure. Upon heat denaturation at 95°C the protein structures, while retaining some helical structure, mostly converted to random coil as indicated by a broad minimum around 203 nm. After lowering the temperature back to 25°C all variants were able to refold to a similar, mostly α-helical structure as before heat denaturation (Supplementary Figure S5). Thermal stability was estimated from heat denaturation curves and the melting temperatures (T_m_) were determined from the half-denaturation points between the native and unfolded states. Dimerization of MaSp NT was shown to increase protein stability through neutralization of intramolecular repelling charge clusters (Kronqvist et al., 2014), which either become protonated or form intermolecular salt bridges. Despite the same number of charged residues (11), the thermal stability of TuSp NT was higher (T_m_ 62°C and 75°C at pH 8 and pH 5.5, respectively) than reported for wt *E. australis* MaSp NT [T_m_ 54°C and 65°C (Kronqvist et al., 2014)] (Figure 5), which is probably due to the presence of a disulfide bond in TuSp NT. TuSp NT_A70E_ showed reduced stability at pH 5.5 (T_m_ 61°C and 67°C at pH 8 and pH 5.5, respectively), in agreement with destabilization of the dimer conformation. TuSp NT_D40RR63D_ showed high stability at both pHs (T_m_ 73°C and 74°C at pH 8 and pH 5.5, respectively), in line with intramolecular salt bridge formation. TuSp NT_E37QE82Q_ showed increased stability at pH 8 (T_m_ 70°C and 74°C at pH 8 and pH 5.5), which confirms elimination of a destabilizing interaction. TuSp NT_E37QE82QE85Q_ and TuSp NT_E37QE82QE85QD130N_ variants showed similar stability at both pHs (T_m_ 69–70°C and T_m_ 70–72°C, respectively), and at the same time were more stable than TuSp NT at pH 8, but less stable at pH 5.5. The decreased stability at pH 5.5 suggests neutralization of residues not involved in the protonation.

## Discussion

We performed structural studies and dimerization analysis of TuSp NT from *A. argentata* using NMR, SEC, and CD experiments, which showed that the pH-dependent stable dimer formation is conserved in tubuliform silk. However, TuSp NT could not be stabilized in the monomer conformation for NMR structure determination regardless of the environmental conditions. Furthermore, introduction of mutations in TuSp NT_K40DD63R_ and TuSp NT_A70E_, corresponding to monomeric and pH insensitive MaSp and FlSp NT variants (Jaudzems et al., 2012; Kronqvist et al., 2017; Sarr et al., 2022), failed to fully stabilize TuSp NT in the monomeric conformation. In order to identify residues that could potentially participate in the pH dependent dimerization mechanism, we determined the solution structure of TuSp NT dimer at pH 5.5. Guided by the insights from the structure, we designed the mutants TuSp NT_E37QE82Q_, TuSp NT_E37QE82QE85Q_, and TuSp_E37QE82QE85QD130N_, in which several clustered and surface-exposed acidic residues were exchanged for their amide counterparts. However, none of them behaved as a constitutive dimer and retained pH sensitivity. SEC and HSQC NMR data showed that the variants assume an intermediate conformation (i.e., with at least one of the titrating carboxylates neutralized) at high pH and are converted into dimeric conformation at pH 5.5. CD experiments showed that thermal stability of the mutants is higher at pH 8, however, not approaching the stability of TuSp NT at pH 5.5.

**FIGURE 6:**
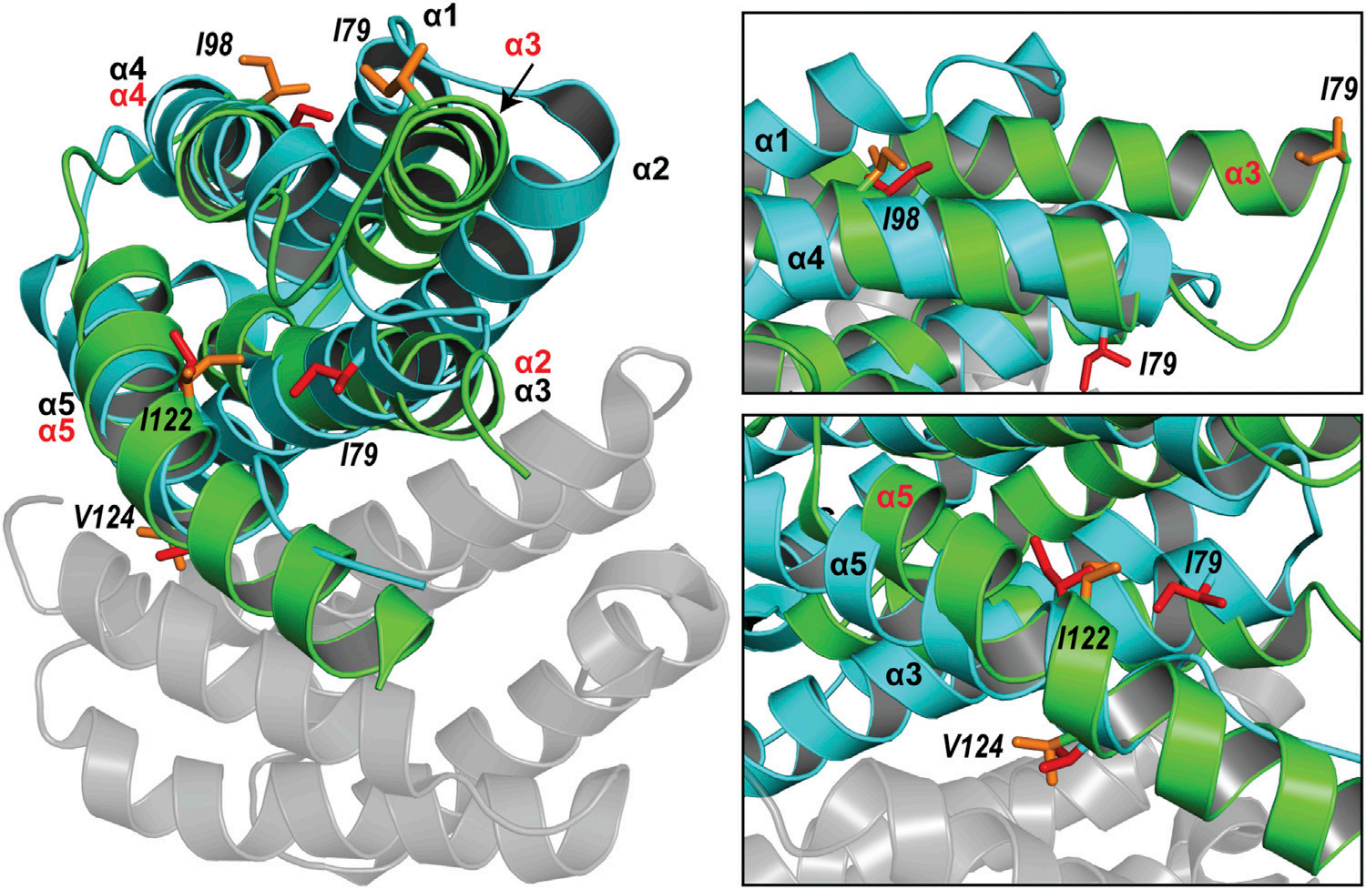
Superposition of the structures of *A. argentata* TuSp NT dimer (PDB ID 6TV5, cyan and gray) and *N. antipodiana* TuSp NT monomer (PDB ID 2K3Q, green). Helices of *A. argentata* TuSp NT are identified with black font, whereas helices of *N. antipodiana* TuSp NT are identified with red font. Hydrophobic residues mutated by Wang et al. (2021) are shown with stick representation in both structures in red (*A. argentata* TuSp NT) and in orange (*A. antipodiana* TuSp NT).

The spidroin NT domains from most spider silk types and species have been shown to adopt a monomeric or dimeric five-helix bundle structure depending on environmental conditions. However, the previously determined TuSp NT structure from *N. antipodiana* shows an atypical four-helix arrangement that is distinct from the five-helix NT structures, raising questions about the fold conservation. Despite high sequence similarity (66% identity), our herein determined TuSp NT structure from *A. argentata* reveals a five-helix composition, which is highly similar to the structures of MaSp, MiSp, AcSp, and FlSp NTs. Superposition of the two TuSp NT structures shows a different organization of the first three helices (Figure 6). The helix α1 in *A. argentata* TuSp NT is replaced by α3 in the *N. antipodiana* structure, α3 is substituted by α2, while helix α2 of the *A. argentata* protein is missing in the *N. antipodiana* structure. Knowing that the first 36 residues of the *N. antipodiana* protein were truncated for the structure determination, the differences are apparently due to a helical reorganization aimed to preserve its hydrophobic core, which is mainly formed by the helices α1 and α3-α5. Besides, the solution conditions used for each structure determination are vastly different—the *N. antipodiana* TuSp NT was studied at neutral pH (50 mM Tris-HCl, pH 7) in presence of 100 mM DPC, whereas we employed acidic conditions (20 mM sodium acetate, 20 mM NaCl, pH 5.5). Accordingly, the *N. antipodiana* structure at pH 7 shows a monomer, whereas the *A. argentata* TuSp NT at pH 5.5 is a dimer. The use of a shorter construct and presence of 100 mM DPC, which according to the authors was necessary to avoid aggregation, raises concerns about the relevance of the *N. antipodiana* structure. Our study shows that the structured part of TuSp NT begins already at residue 10, both for the wt protein at pH 5.5 (Figure 3) as well as the A70E mutant at pH 8.0 (Supplementary Figure S2). Hence, the complete first α-helix (residues S12-I27) and beginning of the second α-helix (residues P31-Q36) is lacking in the *N. antipodiana* TuSp NT structure. Several hydrophobic residues as I80 and I98 showing surface localization in the *N. antipodiana* structure, which have been suggested to play a role in the intermolecular association of TuSp NT (Wang et al., 2021) are buried in the hydrophobic core in our structure (Figure 6). Additionally, replacement of the α1-α4 helical interface by α3-α4 as in the *N. antipodiana* structure would not be possible in a full-length construct, because C22 from α1 forms a disulfide bond with C102 from α4, placing these helices next to each other. Altogether, the newly obtained results indicate that, under physiological conditions, full-length TuSp NT adopts a five-helix bundle structure as the other NTs.

**FIGURE 7:**
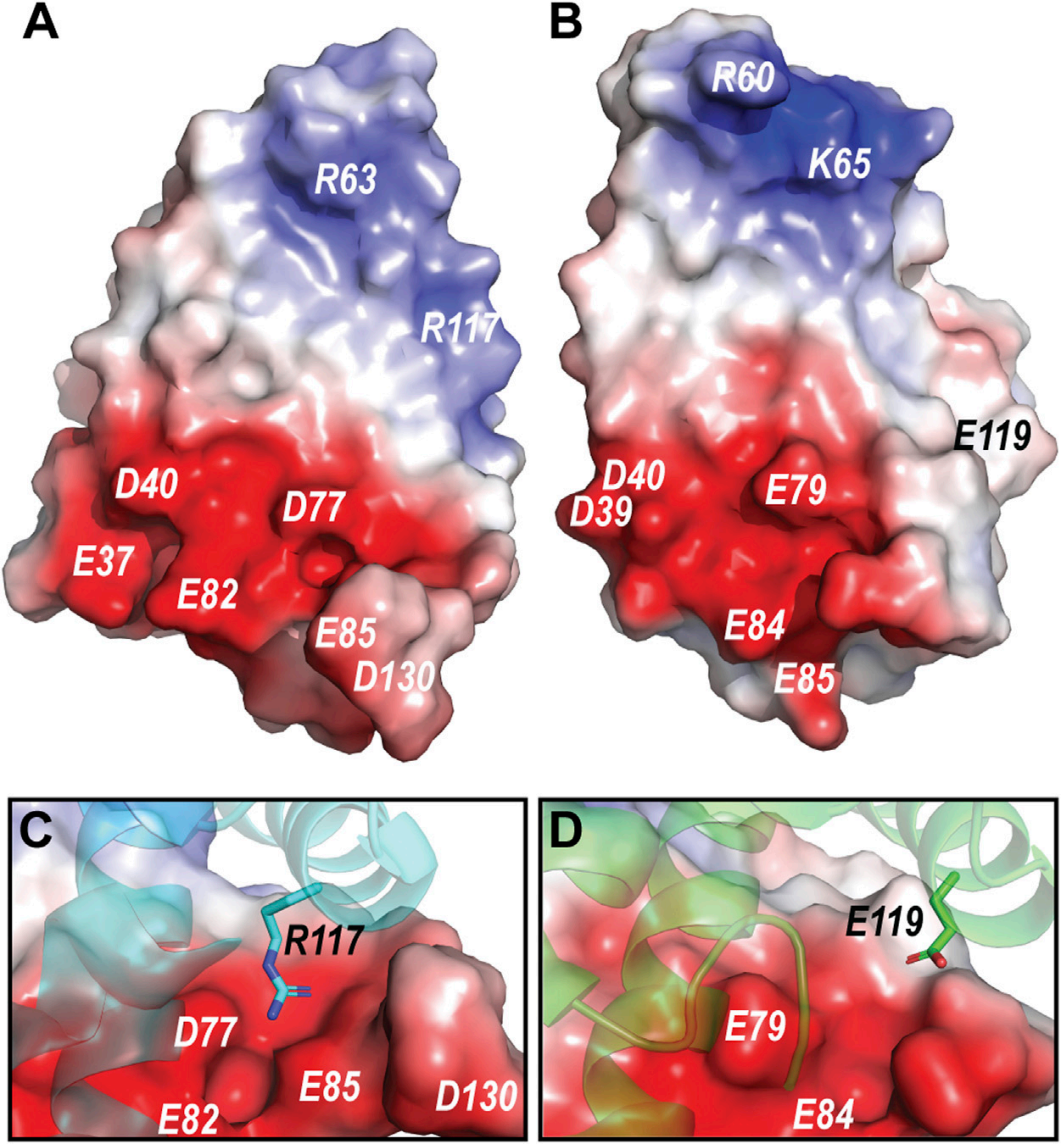
**(A)** Electrostatic potential of *A. argentata* TuSp NT dimer subunit. **(B)** Electrostatic potential of *E. australis* MaSp NT (PDB ID 2LTH) dimer subunit. Red color indicates negative charges, and blue color indicates positive charges. The surface-exposed charged residues are labeled. **(C)** and **(D)** shows contact surfaces of the residues R117 and E119 from the opposite subunit at the TuSp NT and MaSp NT dimer interfaces, respectively.

The pH dependent dimerization of MaSp and MiSp NT was found to be mediated by sequential protonation of three specific glutamic acid residues (E79, E84, E119 in MaSp and E73, E76, E115 in MiSp NT). In the *A. argentata* TuSp NT, only E84 (E82 in TuSp NT) is structurally preserved, whereas E79 and E119 are replaced by D77 and R117, respectively, and form an intermolecular salt bridge in the dimer structure. These substitutions alter the electrostatic surface potential of each monomer subunit, making the positively charged pole more extensive (i.e. spread out) than in MaSp NT (Figure 7), which could facilitate intermolecular association. Additionally, the charge repulsion between two glutamate residues at the dimer interface is abolished potentially allowing the subunits to associate before the protonation events. This could explain why we were unable to stabilize the monomeric conformation of TuSp NT for NMR structure determination. Besides these charge interactions, pre-arrangement of α-helices has been suggested to be important for the NT dimerization. In MaSp NT the helical reorganization is facilitated by swinging out of the wedged W10 residue and subsequent repacking of the hydrophobic core. In TuSp NT W10 is replaced by F10, which is buried in the hydrophobic core of the dimer structure. This is similar to FlSp NT, for which the monomer and dimer structures showed the same buried F10 side chain orientation (Sarr et al., 2022). Thus, the structural reorganization during TuSp NT dimerization does not seem to require relocation of F10 side chain. Heiby et al. (2019) reported that the unusually high content of methionines within the core region of *E. australis* MaSp NT plays an important role in the monomer-dimer structural transition. Exchange of the core methionines with the bulkier leucines made the monomeric MaSp NT more rigid, which abolished the movement of its helices and ability to form a dimer. The lack of the tryptophan and methionine residues in TuSp NT sequence (Figure 1) could abolish its hydrophobic core plasticity, locking the core in a dimer-like conformation also at neutral pH, albeit with different dynamics. Instead, the monomer-dimer structural rearrangement likely involves slight movement of helices due to neutralization (protonation) of repulsing charges across intramolecular helical interfaces.

Since E82 shows a similar side chain orientation to E84 in MaSp NT and additionally has E37 located in close proximity, we hypothesized that neutralization of these two residues may be important for establishing the characteristic handshake interaction between E82 and D40 as seen in the MaSp NT dimer. Although E37 is not conserved in most other spidroins, it is highly conserved between TuSp NTs from different species (Supplementary Figure S6). However, analysis of the double mutant TuSp NT_E37QE82Q_ showed that some other residue is additionally protonated at low pH. The only other well conserved glutamate residue among TuSp NTs is E85, which in some conformers of our structure is spatially close to D130 of the same subunit and the charge clash could prevent it from assuming a dimer-compatible conformation. However, preparation and characterization of the triple and quadruple mutants TuSp NT_E37QE82QE85Q_ and TuSp_E37QE82QE85QD130N_ gave similar results as for TuSp NT_E37QE82Q_. This result does not rule out the involvement of at least some of the mutated residues in the observed pH sensitivity, because the studied combinations may bear disruptive mutations affecting the pK_a_ of other residues that participate in the protonation events of the wt protein.

In summary, our obtained results clearly indicate that the mechanism of pH-dependent dimerization is different for TuSp NT than it is for MaSp and MiSp NT. We show that in contrast to previous findings TuSp NT has the same five-helix fold of other NTs and forms a stable dimer at low pH. However, its unique amino acid sequence does not allow full stabilization of the monomer conformation at the conditions, where MaSp NT forms stable monomers (pH 7.2 in presence of 300 mM NaCl). This may be linked to the lack of a well-defined spidroin storage sac as well as different physiological conditions (especially, pH gradient) in the tubuliform glands, which remain to be characterized. Furthermore, the pH dependent stable dimer formation that takes place in the silk duct upon fiber formation involves a very different set of amino acid residues as compared to MaSp and MiSp NT. Further research is needed to identify the exact amino acid residues that become protonated in the TuSp NT dimer at low pH.

## Data Availability

The datasets presented in this study can be found in online repositories. The names of the repository/repositories and accession number(s) can be found below: http://www.wwpdb.org/, 6TV5 https://bmrb.io/, 34473.
