# Supplementary material for: Solution Structure of Tubuliform Spidroin N-Terminal Domain and Implications for pH Dependent Dimerization
Source: Front Mol Biosci. 2022 Jun 14;9:936887. doi: 10.3389/fmolb.2022.936887 (PMC9237525; doi:10.3389/fmolb.2022.936887)
Supplement: Supplementary file 1 [file DataSheet1.PDF]

Supplementary Material

1.1 Supplementary Figures

A

```
MSDKIIHLTD DSFDTDVLKA DGAILVDFWA EWCGPCKMIA PILDEIADEY QGKLTVAKLN

IDQNPGTAPK YGIRGIPTLL LFKNGEVAAT KVGALSKGQL KEFLDANLAG SGSGHMHHHH

                                                          1    6
HHSSGLVPRG SGMKETAAAK FERQHMDSPD LGTDDDDKAM GIHHHHHHEN LYFQGAVTAV
     thrombin                      enterokinase       TEV protease
        16         26         36         46         56         66
PSVFSSPNLA SGFLQCLTFG IGNSPAFPTQ EQQDLDAIAQ VILNAVSTNT GATASARAQA

        76         86         96        106        116        126
LST[A/E]LASSLT DLLIAESAES NYNNQLSELT GILSNCFIQT TGSDNPAFVS RIQSLISVLS

       136
QNTDVNIIST A
```

B

```
               1   5         15         25         35         45
MGIHHHHHHE NLYFQGAVTA VPSVFSSPNL ASGFLQCLTF GIGNSPAFPT Q[E/Q]QQ[D/R]LDAIA
              TEV
        55  protease 65         75                    95        105
QVILNAVSTN TGATASA[R/D]AQ ALSTALASSL T[D/N]LLIA[E/Q]SA[E/Q] SNYNNQLSEL TGILSNCFIQ

       115        125        135
TTGSDNPAFV SRIQSLISVL SQNT[D/N]VNIIS TA
```

Supplementary Figure 1. Amino acid sequences of the studied *A. argentata* TuSp NT expression constructs in **(A)** pET28(a+) and **(B)** pET32(a+) plasmids. Mutations made to obtain constitutive monomer and dimer variants are colored in purple and yellow, respectively. Thioredoxin tag is blue, his-tags are red, S-tag is green, protein fragment after cleavage with TEV protease is bold and protease cleavage sites are underlined.

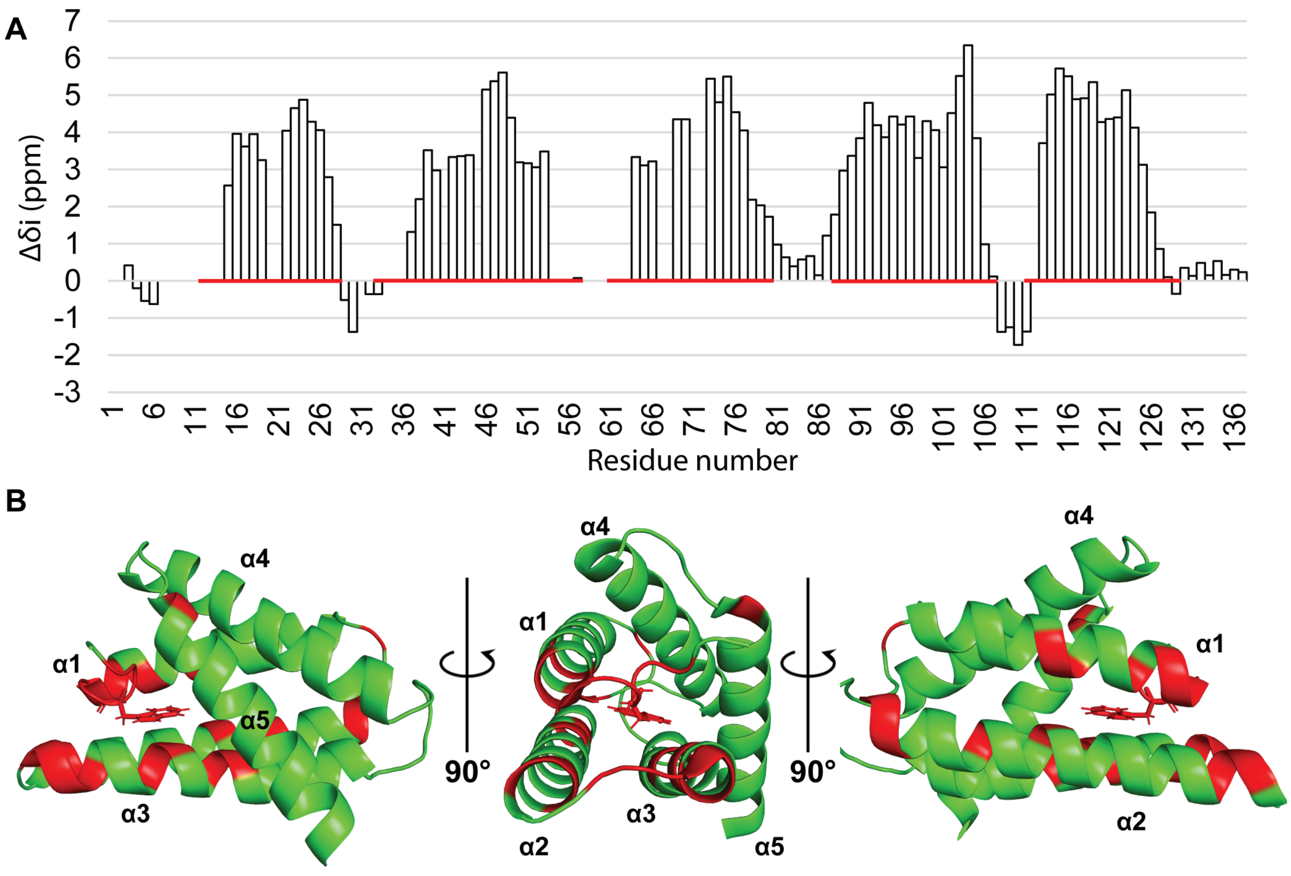

Supplementary Figure 2. **(A)** Secondary chemical shift analysis of TuSp NT_{A70E} mutant in 20 mM NaPi, 300 mM NaCl, pH 7.2. Here, $\Delta\delta_i$ is calculated as $(\Delta C_\alpha - \Delta C_\beta) + (\Delta C_{\alpha-1} - \Delta C_{\beta-1}) + (\Delta C_{\alpha+1} - \Delta C_{\beta+1}) / 3$. $\Delta\delta_i$ values above 1 for three or more consecutive residues are indicative of alpha helix and below −1 of beta sheet secondary structure, whereas $\Delta\delta_i$ values between −1 and 1 indicate non-regular structure. The red line on x axis indicates the locations of alpha helices in *E. australis* MaSp NT monomer (PDB ID 2LPJ). **(B)** Mapping of the unassigned residues on the MaSp NT monomer structure at pH 7.2. Assigned residues are colored in green and unassigned residues are red. W10 is shown with stick representation.

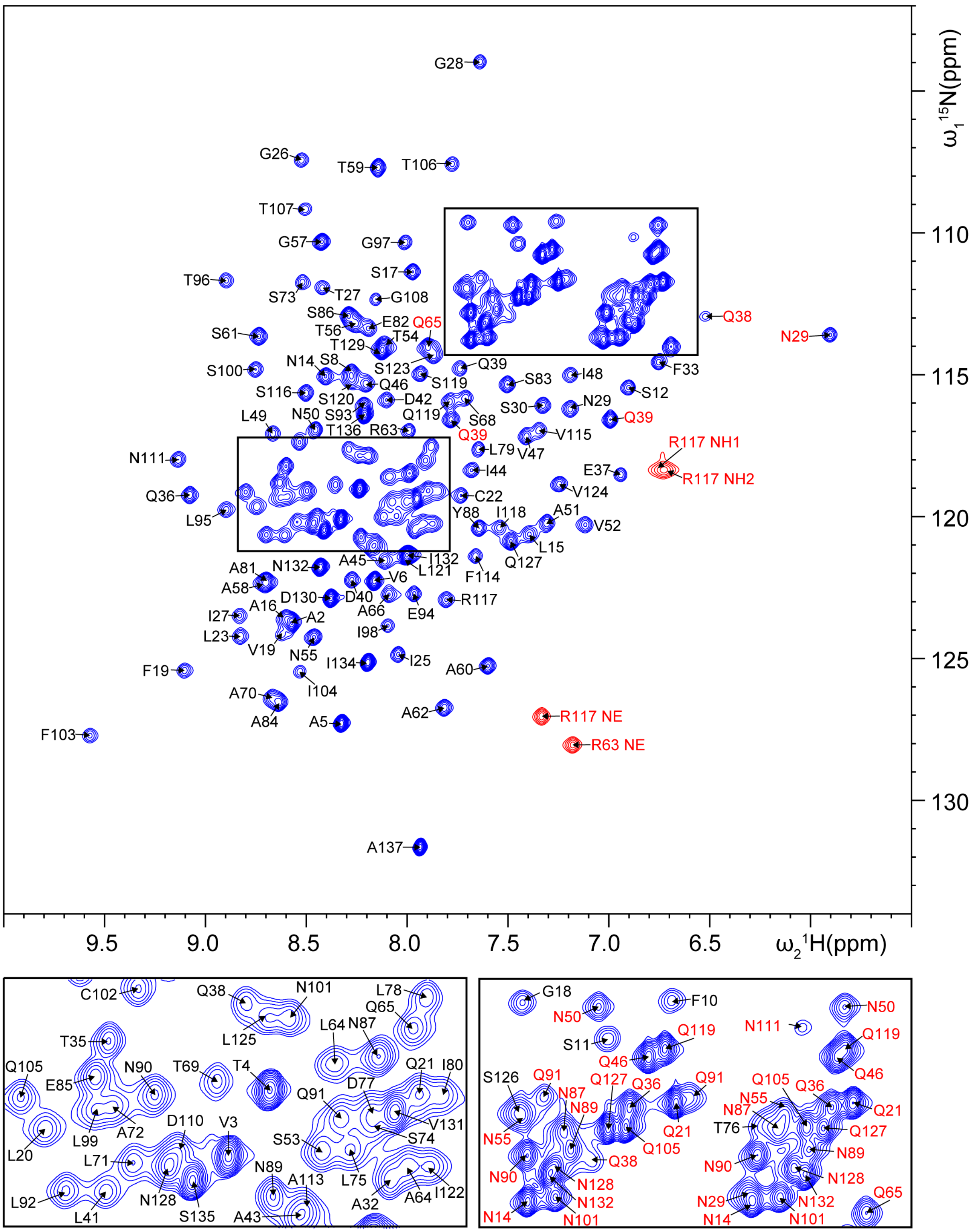

Supplementary Figure 3. Assigned ^{15}N-^{1}H HSQC NMR spectrum of TuSp NT dimer in 20 mM NaOAc 20 mM NaCl, pH 5.5. Backbone and side chain amide resonance assignments are indicated with black and red one letter symbol and residue number, respectively.

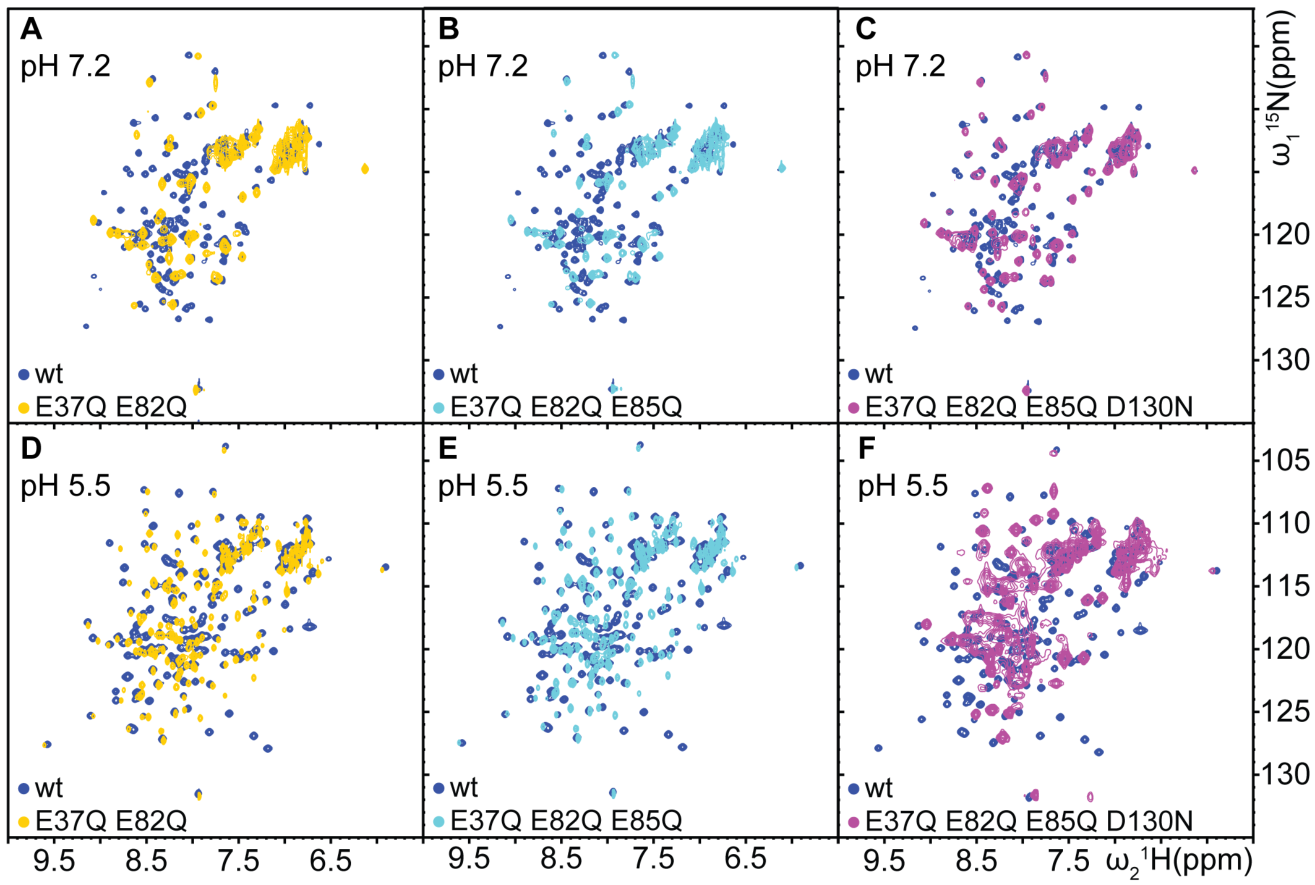

Supplementary Figure 4. Comparison of ^{15}N-^{1}H HSQC spectra of TuSp NT (blue) and its potential constitutively dimeric mutants TuSp $NT_{E37QE82Q}$ (yellow, **A** and **D**)**,** TuSp $NT_{E37QE82QE85Q}$ (cyan, **B** and **E**) and TuSp $NT_{E37QE82QE85QD130N}$ (purple, **C** and **F**) in 300 mM NaCl, 20 mM NaPi, pH 7.2 and 20 mM NaCl, 20 mM NaOAc, pH 5.5 respectively.

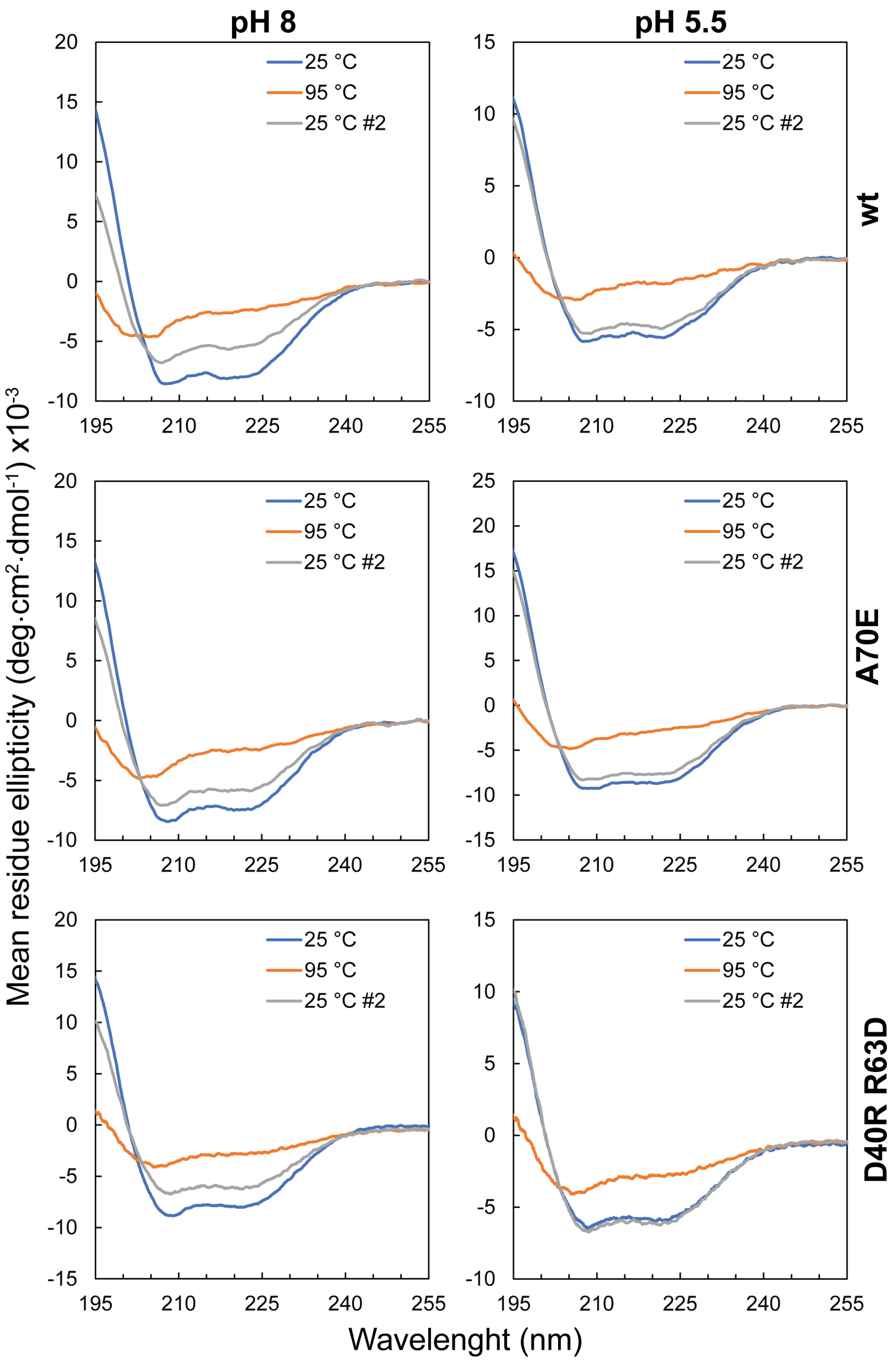
pH 8
pH 5.5
wt
A70E
D40R R63D
25 °C
95 °C
25 °C #2
Mean residue ellipticity (deg·cm²·dmol⁻¹) x10⁻³
Wavelenght (nm)

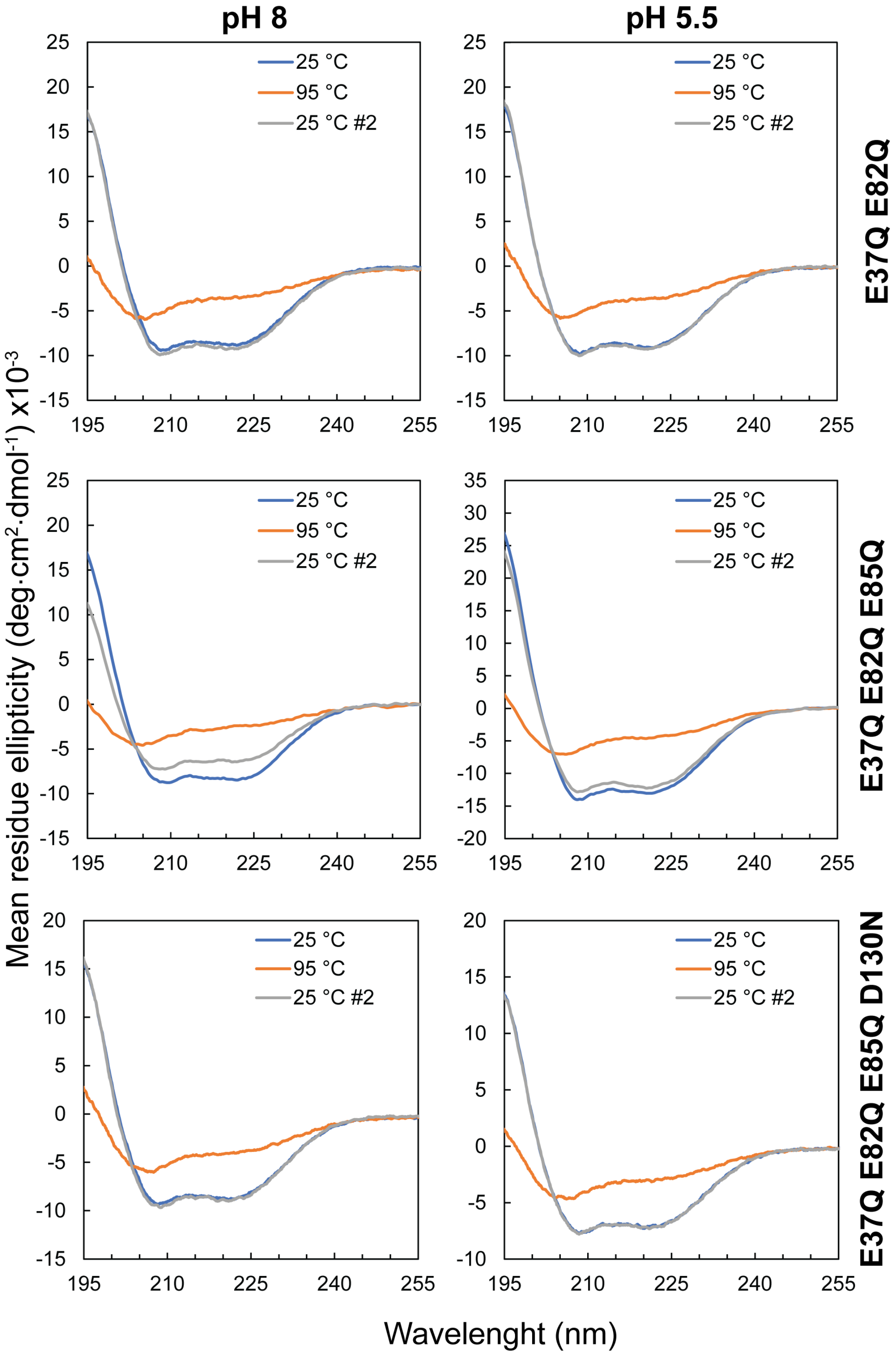

pH 8
pH 5.5
E37Q E82Q
E37Q E82Q E85Q
E37Q E82Q E85Q D130N
25 °C
95 °C
25 °C #2
Mean residue ellipticity (deg·cm²·dmol⁻¹) x10⁻³
Wavelenght (nm)

Supplementary Figure 5. Refolding capacity of TuSp NT, TuSp NT_{A70E}, TuSp $NT_{D40RR63D}$ TuSp $NT_{E37QE82Q}$, TuSp $NT_{E37QE82QE85Q}$ at pH 8.0 and pH 5.5. Each panel shows CD spectra recorded at 25 °C, after denaturation at 95 °C and after refolding at 25 °C.

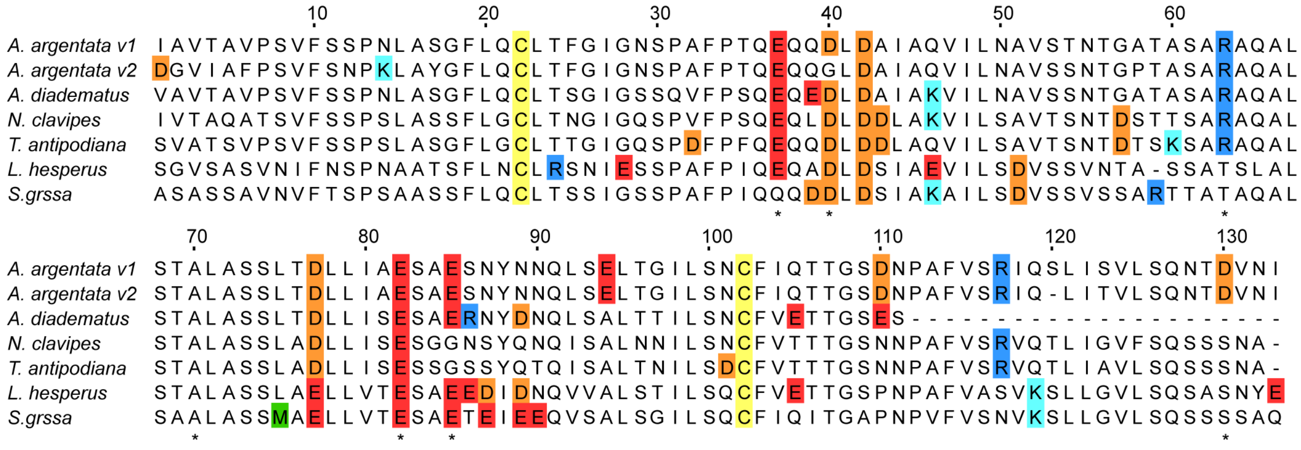

Supplementary Figure 6. Sequence alignment of TuSp NTs from Uniprot data base. Cysteines are shaded in yellow, glutamic acids are red, aspartic acids are orange, arginines are blue, lysines are cyan and methionines are green. The residue positions mutated in our study are marked with stars below the alignment.
